# Complex by design: Hydrotrope-induced micellar growth in deep eutectic solvents

**DOI:** 10.1016/j.jcis.2020.07.077

**Published:** 2020-07-19

**Authors:** Adrian Sanchez-Fernandez, Anna E. Leung, Elizabeth G. Kelley, Andrew J. Jackson

**Affiliations:** a Food Technology, Engineering and Nutrition, Lund University, Box 124, 221 00 Lund, Sweden; b European Spallation Source, Box 176, 221 00 Lund, Sweden; c The NIST Center for Neutron Research, National Institute of Standards and Technology, Gaithersburg, MD 20899-8562, USA; d Division of Physical Chemistry, Lund University, Box 124, 221 00 Lund, Sweden

**Keywords:** Deep eutectic solvent, Worm-like micelle, Hydrotrope, Small-angle neutron scattering

## Abstract

**Hypothesis:**

The self-assembly of ionic surfactants in deep eutectic solvents has recently been demonstrated, opening up new possibilities in terms of the development of formulated products and templating of nanostructured materials. As it occurs in an aqueous environment, the solvophobic effect drives the formation of micelles in these solvents and specific-ion interactions alter the resulting structures. We hypothesized that the presence of hydrotropic salts would greatly affect the micellar structure in deep eutectic solvents, ultimately leading to the formation of worm-like aggregates.

**Experiments:**

A systematic investigation performed on hydrotrope-surfactant assemblies in neat and hydrated 1:2 choline chloride:glycerol is presented. The effect of choline salicylate on the micellization of hexadecyltrimethylammonium chloride at different hydrotrope-to-surfactant ratios was probed by contrast variation small-angle neutron scattering.

**Findings:**

Here the first investigation on salt-induced micellar growth in deep eutectic solvents is presented. The microscopic characterization of the system shows that the micelle-hydrotrope interaction in pure and hydrated deep eutectic solvents results in a significant increase in micelle elongation. The condensation of the hydrotrope on the micelle, which alters the effective monomer packing, leads to the formation of worm-like micelles with tunable morphology and flexibility. The results presented here present new possibilities in terms of self-assembly and co-assembly in neoteric solvents, where micelle morphology can be controlled through surfactant-salt interactions.

## Graphical Abstract

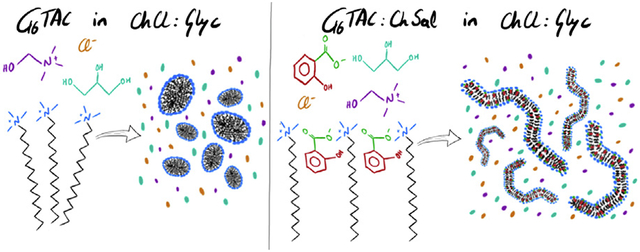

## 1. Introduction

The self-assembly of surfactants into polymer-like structures, namely worm-like micelles (WLM), in water has been studied since the mid-80s. These structures are characterized by their elongation and flexibility, which is a consequence of the minimization of the spontaneous curvature of the assembly for structures with very large aggregation numbers [1,2]. The formation of this class of structure has been reported for different types of surfactants: anionic, cationic, non-ionic, zwitterionic and other complex surfactants (e.g. gemini surfactants or photosurfactants) [2-7]. In particular, WLM comprised of ionic surfactants can be formed through the addition of salts to decrease the charge density in the headgroup region, inducing micelle growth [8,9]. Hydrotropic salts provide a specific case of salt-induced growth at much lower salt concentration, where the charged moiety promotes charge screening and the hydrophobic domain of the salt penetrates the micelle core, thus synergistically modifying surfactant packing [10]. Above a certain surfactant concentration, WLM entangle into dynamic networks and confer interesting rheological properties upon the system, such as shear-thinning and viscoelasticity [11]. These systems have attracted significant interest from fundamental (e.g. dynamics of breakable polymers) and applied research (e.g. rheological modifiers for personal care and drug products) [2,4,12]. The vast majority of this research has focused on aqueous systems. To date, few reports have taken advantage of emerging green solvents to tune WLM formation and growth.

Type III deep eutectic solvents (DES) are one promising class of green solvents obtained through the complexation of a halide salt with an organic hydrogen bond donor at a certain mole ratio. Combinations of precursors allow a myriad of possibilities to be obtained in terms of physicochemical properties of the solvent, enabling tuning for particular applications [13]. They are also readily available, non-toxic and cheap; valuable characteristics in sustainable technologies. The formation and stability of DES have been shown to rely on an extensive hydrogen bond network between their constituents [14]. Due to these characteristics, together with their mild and green character, DES have been proposed as alternative to molecular organic solvents for a variety of applications [15]. Furthermore, their stability in water opens up new possibilities in the control of the solvent properties through the formation of environments with intermediate properties between DES and water [16,17].

It has recently been demonstrated that DES support the self-assembly of anionic, cationic, zwitterionic, and novel surfactants (e.g. ionic liquid-based) in the absence of water [18-22]. The self-assembly of surfactants in (hydrophilic) DES was shown to be driven by the solvophobic effect, similar to the entropy-directed process that occurs in water, polar organic solvents, and ionic liquids [18,20,23,24]. The resulting micelle morphology in DES has been shown to depend on both the surfactant and solvent characteristics, where two main routes can be differentiated: non-interacting and interacting systems. On one hand, non-interacting systems are characterized by the absence of strong electrostatic correlations between surfactant and solvent ions, resulting in a similar behavior to that in water [20]. On the other hand, the charge density of the micelle in interacting systems is affected by the ionic components of the solvent, together with the native surfactant counterion [25,26]. In the latter case, the formation of micelles with different morphologies to those in water and other molecular solvents (e.g. formamide or glycerol) is observed [24,27,28]. Therefore, the self-assembly of a surfactant can be tuned through changes in the physicochemical properties of the solvent, opening up sustainable alternatives for templating of nanostructured materials, the formation of non-aqueous responsive materials, and formulation of gelled products. Furthermore, the presence of water has been shown to affect surfactant self-assembly in DES and adds an extra possibility in terms of controlling the characteristics of the system [25,26]. The present work takes advantage of these tunable features of DES through the addition of a hydrotropic salt to manipulate and control the formation of WLM.

## 2. Experimental methods

### 2.1. Materials

Choline chloride (ChCl, Sigma-Aldrich, 99%), glycerol (Glyc, Sigma-Aldrich, >99%), choline chloride-d_9_ (d-ChCl, Cambridge Isotope Laboratories, N,N,N-trimethyl-d9, 99%, 98% D), glycerol-d_8_ (d-Glyc, Cambridge Isotope Laboratories, 99%, 99% D), choline acetate (ChAc, IoLiTec, 98%), sodium salicylate (NaSal, VWR, >99%) and sodium hydroxide (Sigma-Aldrich, >99%) were used without further purification. All solvent and salt precursors were vacuum dried at 50 °C before use. Protiated or deuterated choline chloride and glycerol were mixed in a 1:2 M ratio and heated to 80 °C until a homogeneous, transparent liquid was formed. The solvents were subsequently equilibrated at 40 °C for 24 h and stored under a dry atmosphere. The hydrated versions of the solvents were prepared by mixing neat DES with water or D_2_O at given mole ratios, and subsequently equilibrated for 24 h. The mole ratios prepared and the equivalent wt% of water for each hydrated solvent are presented in the Supplementary Data (Table S1).

Hexadecyltrimethylammonium chloride (C_16_TAC or h-C_16_TAC, Sigma-Aldrich, 98%) was used as received. Sodium salicylate-d_4_ (d-NaSal, 90% D) was prepared through the deuteration of salicylic acid using a modification of the procedure reported by Sawama et al. [29] and subsequent preparation of the sodium salt. Choline salicylate (h-ChSal) and choline-d_9_ salicylate-d_4_ (d-ChSal) were prepared at the Deuteration and Macromolecular Crystallisation DEMAX facility (ESS, Sweden) using a modification of the protocol presented by Kukawka et al. [30]. The deuteration level of these compounds was assessed by electrospray ionisation mass spectroscopy. Details of the synthesis and characterisation of these compounds are presented in the Supplementary Data.

Contrast variation small-angle neutron scattering (SANS) was used to investigate the structure of the micelles formed in DES. Two different isotopic mixtures of the system were prepared using different isotopologues. Samples were prepared at different surfactant and salt solutions by diluting the stock solutions as follows: protiated surfactant (h-C_16_TAC) with protiated salt (h-ChSal or h-ChAc) in deuterated solvent (d-ChCl:d-Glyc), and protiated surfactant (h-C_16_TAC) with deuterated salt (d-ChSal) in deuterated solvent (d-ChCl:d-Glyc).

### 2.2. Methods

#### 2.2.1. Small-angle neutron scattering

Contrast variation SANS measurements were performed on vSANS at NIST Center for Neutron Research (US). This instrument uses a multiple detector array, of which front and middle carriage were used at 4.5 m and 18 m respectively for the low momentum transfer (q) range data and a second configuration with the detector carriages at 1 m and 5 m for the high q data. A 6.7 Å neutron wavelength (dλ/λ = 12%) was used with two different configurations of neutron guides, 0 and 9 for the low and high q range configurations, respectively. These configurations provided a momentum transfer (q) range of 0.0023 Å^−1^ to 0.36 Å^−1^. Data reduction was performed using the standard protocols of the beamline and data were corrected for detector efficiency, background noise, sample transmission, and scattering from an empty cell. The scattering of the solvent was subsequently subtracted accounting for the incoherent contribution of each sample using SasView 4.2.2 [31]. The output data were absolute intensity [I(q), cm^−1^] versus momentum transfer [q, Å^−1^].

Samples were loaded in 1 mm path length, 10 mm width, quartz Hellma cells and placed in a temperature-controlled sample changer at 50 °C. This temperature was selected in order to maintain the system above its Krafft point and to allow comparison with previous investigations of analogous systems [20,26].

#### 2.2.2. Data analysis

The scattering data from centrosymmetric, uniform colloidal systems may be generalised using the equation:

$$I(q)=NV(\Delta SLD{)}^{2}P(q)S(q)+B$$

where N and V are the number and volume of particles, and ΔSLD is the difference in scattering length density (SLD) between the solvent and the particles. P(q) is the form factor and corresponds to the intraparticle contribution to the scattering, thus it depends on the particle morphology. S(q) corresponds to the structure factor and relates to the interparticle interference in the scattering signal. Finally, B is a q independent factor that accounts for the background signal. As the SANS data are normalised to absolute intensities, the volume fraction of scatterers (φ) can be extracted from the fits. The neutron SLD of each component of the system was calculated from the neutron scattering length of the unit (b) and the volume it occupies (V_m_). These values are presented in the Supplementary Data (Table S2).

SANS data were analysed using a model-based fitting approach, which uses mathematical models to determine the scattering from given particle morphologies, P(q), and interparticle interactions, S(q). Considering that micellar growth in DES normally occurs in the perpendicular axis to the rotational axis of the micelle [25], different morphologies can be differentiated depending on the degree of elongation. For shorter micelles (elongation ≤ 3 times the radius of the cross-section), a uniform prolate ellipsoid model was found to appropriately describe the scattering from the shorter aggregates [20,32]. This P(q) model uses two structural parameters: the equatorial radius, parallel to the rotational axis of the micelle (r), and the polar radius, perpendicular to the rotational axis of the micelle (r_po_). Upon the addition of the hydrotrope to the system, micellar growth is observed. When the elongation of the micelles increases (elongation > 3 times the radius of the cross-section), it was found that a uniform cylinder model was more appropriate to describe their morphology. The structural parameters that this model uses to describe micelle morphology are the cross-sectional radius (r) and the contour length (L).

As micelles grow in length, it becomes less entropically favourable to remain as stiff bodies and, in this regime, they are better described as semiflexible cylinders [2,12,33,34]. These self-assembled structures, namely worm-like micelles, are composed of a succession of rigid sections interconnected by flexible nodes. The small-angle signal from these scatterers is characterised by an asymptotic behaviour defined by three different regions in reciprocal space: the low q Guinier region that relates to the micelle length (q < 0.003 Å^−1^); intermediate q shoulder which arises from the persistence length of the micelle (0.003 Å^−1^ < q < 0.01 Å^−1^); and the high q decay that corresponds to the signal from the micelle cross-section (q > 0.01 Å^−1^). Thus, the structural features of these very long bodies (elongation > 50 times the radius of the cross-section) can be described using three parameters: the micelle contour length (L), the persistence length (l_p_) and the cross-sectional radius (r); where l_p_ relates to the statistical length of the rigid segments within the micelle [12,32].

**Fig. 1.**
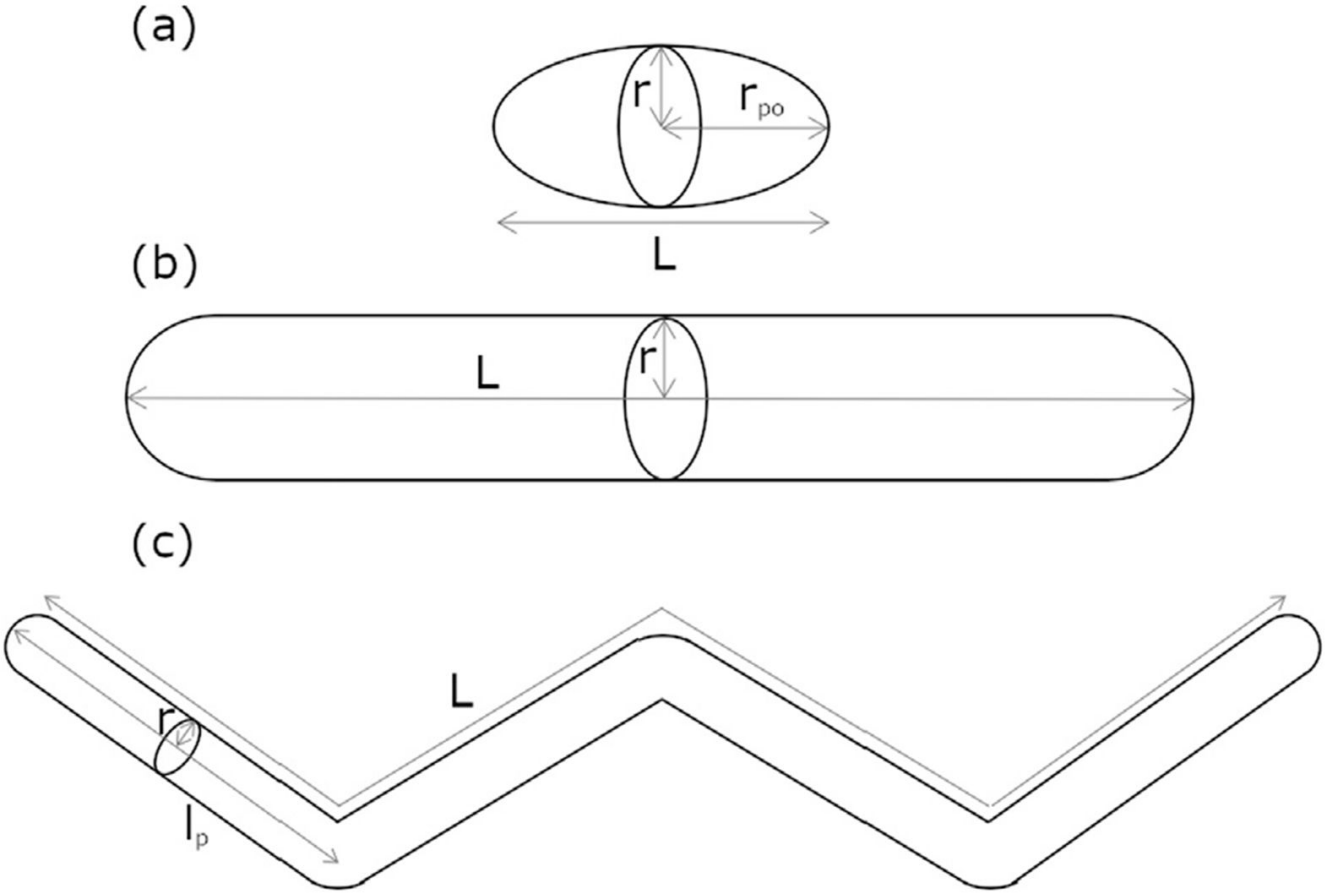
Schematic representation of the structures used to describe micelle morphology: (a) prolate ellipsoid, (b) cylinder, and (c) semiflexible cylinder. The structural parameters used to describe the size of the shapes are included in the figure.

To allow direct comparison between the different models used here, the elongation obtained through the ellipsoid modelling was converted to L as L = 2r_po_. A schematic representation of the structural models used in the data analysis is presented in Fig. 1.

Previous investigations of surfactant self-assembly in DES did not account for micellar polydispersity [20,26], as globular micelles tend to show narrow size distributions and, thus, to be relatively monodisperse. Nonetheless, Mukerjee demonstrated that large asymmetric micelles in thermodynamic equilibrium tend to show aggregation number indexes (M_w_/M_n_) around 2, therefore, showing wide size distributions in length [35]. To account for such polydispersity effects in contour length and radius of the cross-section, polydispersity functions were included. The size distributions were represented using a Schulz function with p = σ/L, where L is the average length of the micelle and σ is the root-mean-square deviation from L [36]. The width of the Schulz distribution is defined by a parameter z such that z=(1-p^2^)/p^2^. For the radius of the cross-section, the size distribution was accounted for by using z = 100 (hence p = 0.1). In order to account for the wide distribution of micelle length (M_w_/M_n_ = 2), z was 1 and thus p = 0.7 [35,36]. A value of p = 0.15 was used to account for the polydispersity in the micelle cross-section. The distribution function was parametrised by using N_pts_ = 160 and N_σ_ = 15. For further reference on the parametrisation refer to the SasView 4.2.2 manual.

The form factor and structure factor models are implemented in SasView 4.2.2 and were fitted to the experimental data by using a Levenberg – Marquardt algorithm [31]. The models were smeared using a Gaussian function at a variable dq/q, which were calculated from the wavelength and geometrical resolution for each q-value in the data reduction procedure, to account for the instrument resolution.

## 3. Results and discussion

### 3.1. Results

**Fig. 2.**
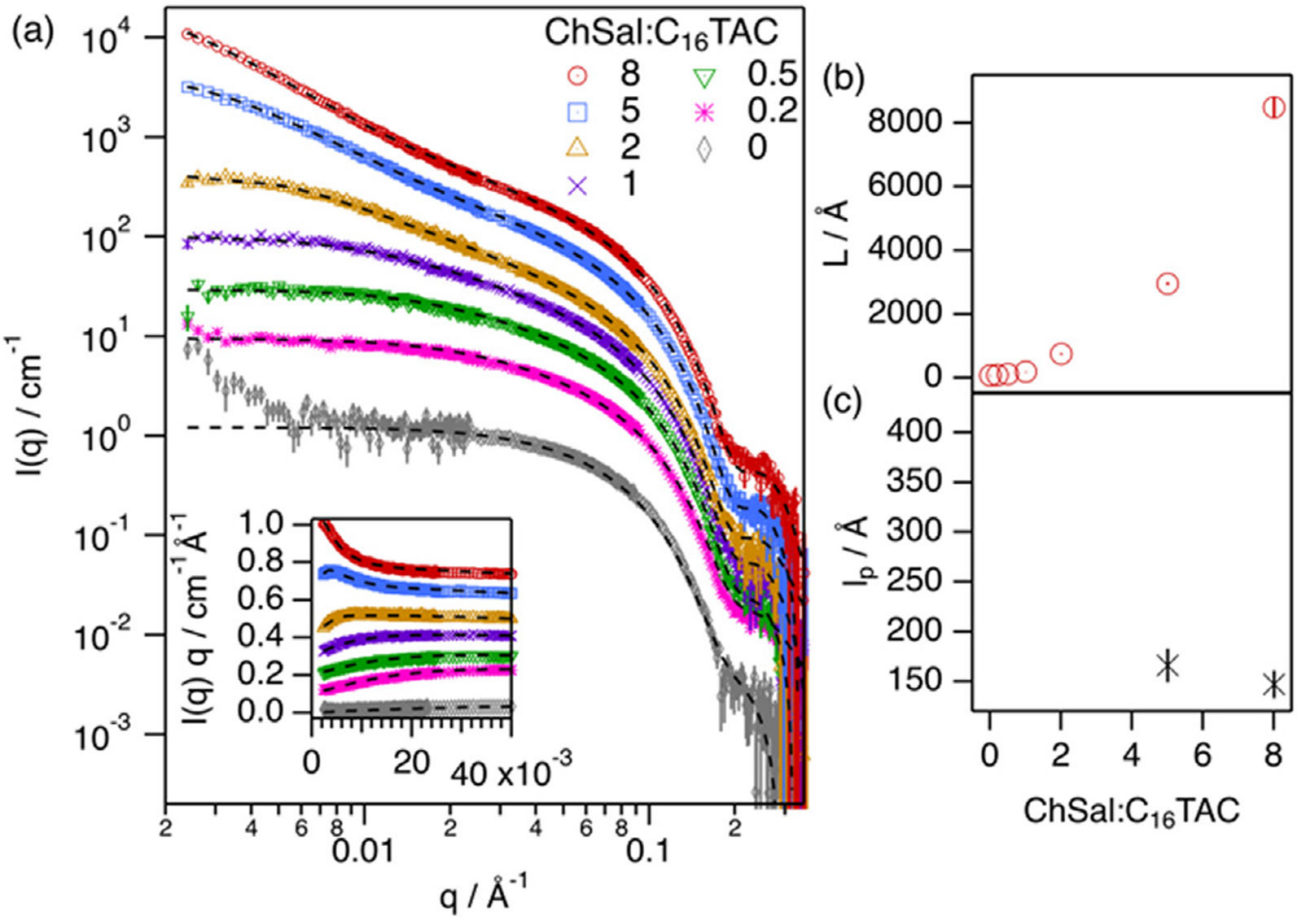
(a) SANS results and best fits of 40 mmol/L (mM) h-C_16_TAC at different h-ChSal/h-C_16_TAC ratios in 1:2 d-ChCl:d-Glyc (as shown in the plot legend), where h and d stand for protiated and deuterated compounds. Measurements were performed at 50 °C on the vSANS instrument at NCNR (US). Fits are presented as black dashed lines. The inset presents the Holtzer plots (I(q) q vs q) of the SANS data presented in (a). Data and fits have been offset for clarity. Structural parameters derived from the model-based analysis using uniform bodies are presented at different h-ChSal/h-C_16_TAC: (b) contour length (L) and (c) persistence length (l_p_) [32]. Due to the limited q-range of the SANS measurements, the largest dimension which could be probed was ≈3000 Å. Thus, values larger than that are an estimation of the elongation that resulted from the fitting approach. All error bars represent one standard deviation and in some cases are smaller than the symbols.

Initially, the effect of a common hydrotropic salt, sodium salicylate, on the behavior of cationic micelles in DES was tested. However, the solubility of this salt was found to be remarkably low. Therefore, it was decided to prepare and use an analogous hydrotropic salt with higher solubility in DES, and it was found that ChSal is very soluble in 1:2 choline chloride:glycerol (ChCl:Glyc) [30]. To determine the structural effect of the hydrotrope on the morphology of the micelles in ChCl:Glyc, SANS experiments were performed. SANS patterns and best fits using model-based analysis (uniform scatterers) are shown in Fig. 2, together with the structural parameters derived from those fits.

The cationic surfactant C_16_TAC in ChCl:Glyc without added ChSal has been found to form globular micelles of similar dimensions to those of hexadecyltrimethylammonium bromide in ChCl: Glyc [20]. The addition of the hydrotropic salt promotes an increase in the micelle contour length (L), even at low salt concentration. At low ChSal/C_16_TAC ratios, between 0.2 and 2, micelles behave as rigid cylinders of larger L than C_16_TAC micelles in the absence of salt. Above a ChSal/C_16_TAC ratio of 2, the formation of the WLM is observed, where the elongation significantly increases with adding more hydrotrope (see Fig. 2b). These trends are similar to those found in water for similar hydrotrope-surfactant systems in the dilute regime [11,33]. Interestingly, significant differences appear between DES and water at ChSal/C_16_TAC ratios close to and above 1, as the system remains in the micellar form in DES but phase-separates in water. The phase-separation is believed to arise from complete charge neutralization between surfactant and salt in water as they co-assemble due to their hydrophobic character. However, some of the hydrotropic salt remains solvated in DES, not interacting with the micelle, and thus not inducing complete charge neutralization of the system at equimolar concentrations.

The presence of micellar persistence length (l_p_), as a quantifier of the micellar flexibility, appears as a shoulder at intermediate q-values in the SANS data. Such a feature is better observed using the Holtzer plot (I(q) q vs q), as this correlation length appears as a broad peak in the curve (See inset of Fig. 2a). At ChSal/C_16_TAC ratios between 0 and 2, the Holtzer plots do not show any peak and thus micelles lack any statistical length. When attempting to fit these data using a flexible cylinder model, these asymmetric assemblies showed an l_p_ larger than L, indicating again that l_p_ could not be extracted. As such, the formation of rigid rods best describes the morphology of the micelles at these ratios. When the ChSal/C_16_TAC is increased beyond 2, a correlation peak is observed at intermediate q (≈0.006 Å^−1^) in the Holtzer plots of the SANS data. When fitting these data with the flexible cylinder model, it is seen that WLM with statistical lengths around 160 Å (Kuhn length = 320 Å) are formed. A geometric analysis of the contributions to micelle flexibility performed by Appell et al. showed that a minimum l_p_ of 90 Å applied for the steric contribution and, when electrostatic effects were added to this, the minimum l_p_ increased to 200 Å [37]. Considering the values observed for the system investigated here, it can be seen that the l_p_ is larger than that for excluded volume interactions but smaller than the one for electrostatically interacting chains. This suggests that whilst the steric contribution is retained, the electrostatic repulsion is partially hindered but not completely suppressed in DES, as previously observed for micellar interactions in a more concentrated regime (> ≈ 150 mM) [20,21,38].

**Fig. 3.**
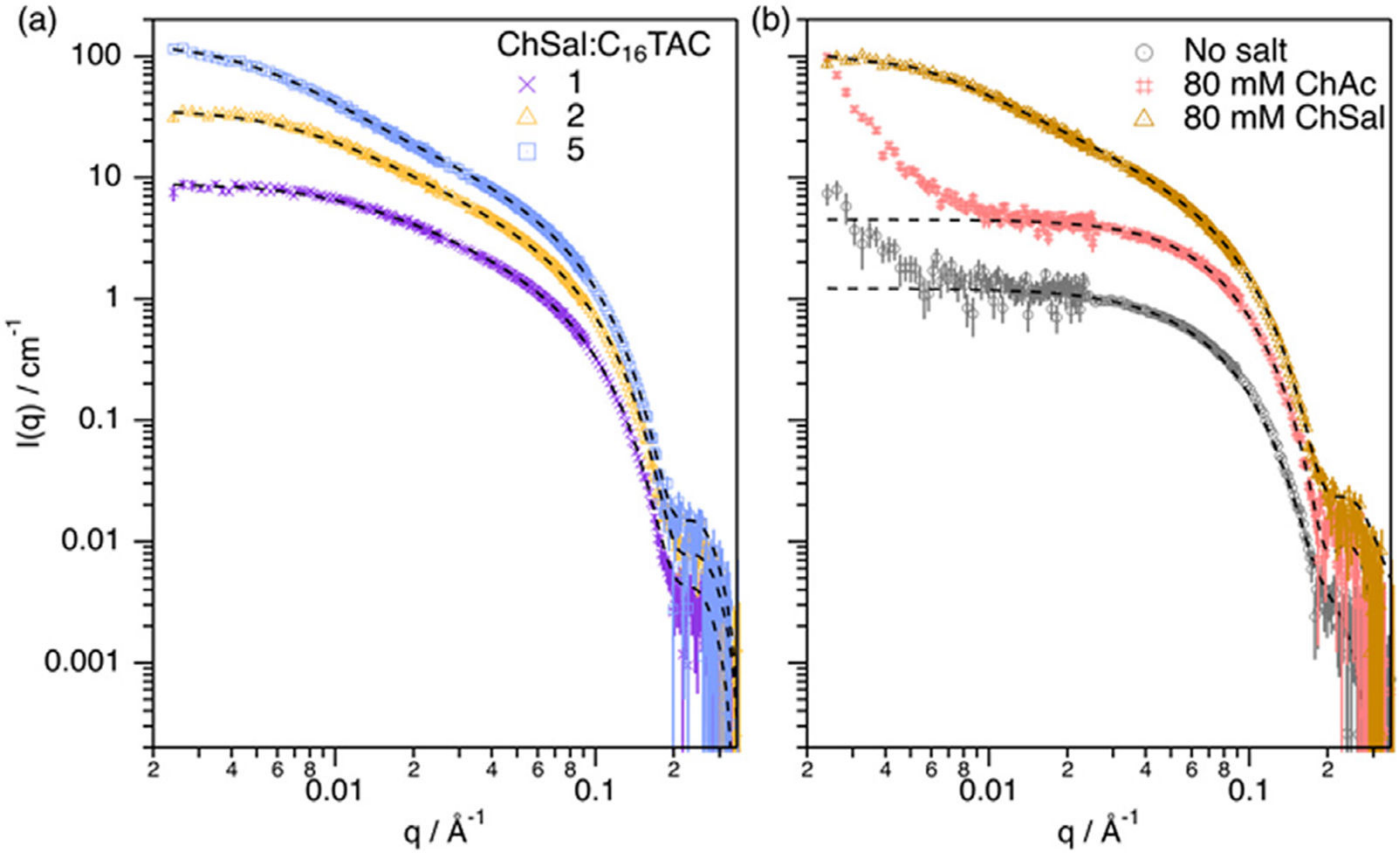
(a) SANS results and best fits of 40 mM h-C_16_TAC at different d-ChSal/h-C_16_TAC ratios in 1:2 d-ChCl:d-Glyc (as shown in the plot legend), where h and d stand for protiated and deuterated compounds. (b) SANS results and best fits of 40 mM h-C_16_TAC with no salt, 80 mM ChAc and 80 mM ChSal in 1:2 d-ChCl:d-Glyc (as shown in the plot legend) where h and d stand for protiated and deuterated compounds. Fits using model-based analysis are presented as black dashed lines. Measurements were performed at 50 °C on the vSANS instrument at NCNR (US). Fits are presented as black dashed lines. Data and fits have been offset for clarity. All error bars represent one standard deviation and in some cases are smaller than the symbols.

To determine the molecular origin of the micellar growth, contrast variation SANS measurements were performed on two isotopic mixtures: h-C_16_TAC, h-ChSal, d-ChCl:d-Glyc and h-C_16_TAC, d-ChSal, d-ChCl:d-Glyc. The SANS data at both contrasts were individually fitted in an attempt to reveal the molecular interaction between the surfactant and the hydrotrope. SANS data and best fits are presented in Fig. 3a. The structural parameters derived from the data analysis are presented in Table S5.

The results from this analysis show that, for this system, the cross-sectional size of the micelles is slightly, but consistently, smaller (between 1% and 5%) for the system containing deuterated hydrotrope. This can be interpreted as follows: the adsorption of the hydrotrope in its deuterated form reduces the scattering contrast between the outer layer of the protiated surfactant micelle and the deuterated solvent, and this would cause the micelle to look smaller than the analogue micelle with the protiated hydrotrope. This suggests that the imbibition of the hydrophobic domain of ChSal into the micelle core is the driving force of the interaction.

In order to corroborate this mechanism, the effect of the addition of choline acetate (ChAc) on micelle structure was investigated. SANS data and best fits using a uniform ellipsoid or cylinder model are presented in Fig. 3b. The structural parameters derived from the data analysis are presented in Table S6. The acetate anion shares a structural similarity with the salicylate anion, the carboxylate group, but it lacks the aromatic ring present in the hydrotrope (See Fig. S13). SANS data of this sample (ChAc/C_16_TAC = 2, 40 mM C_16_TAC) were consistent with the formation of globular aggregates with very similar dimensions to the micelles in the absence of salt. This similarity is indicative of a significantly weaker ion condensation from ChAc compared to ChSal (r = 21.7 ± 0.4 Å, L = 68.6 ± 0.8 Å). As such, the presence of a hydrophobic domain in the salt is required to promote a greater ion condensation and micelle growth in DES at this salt concentration.

**Fig. 4.**
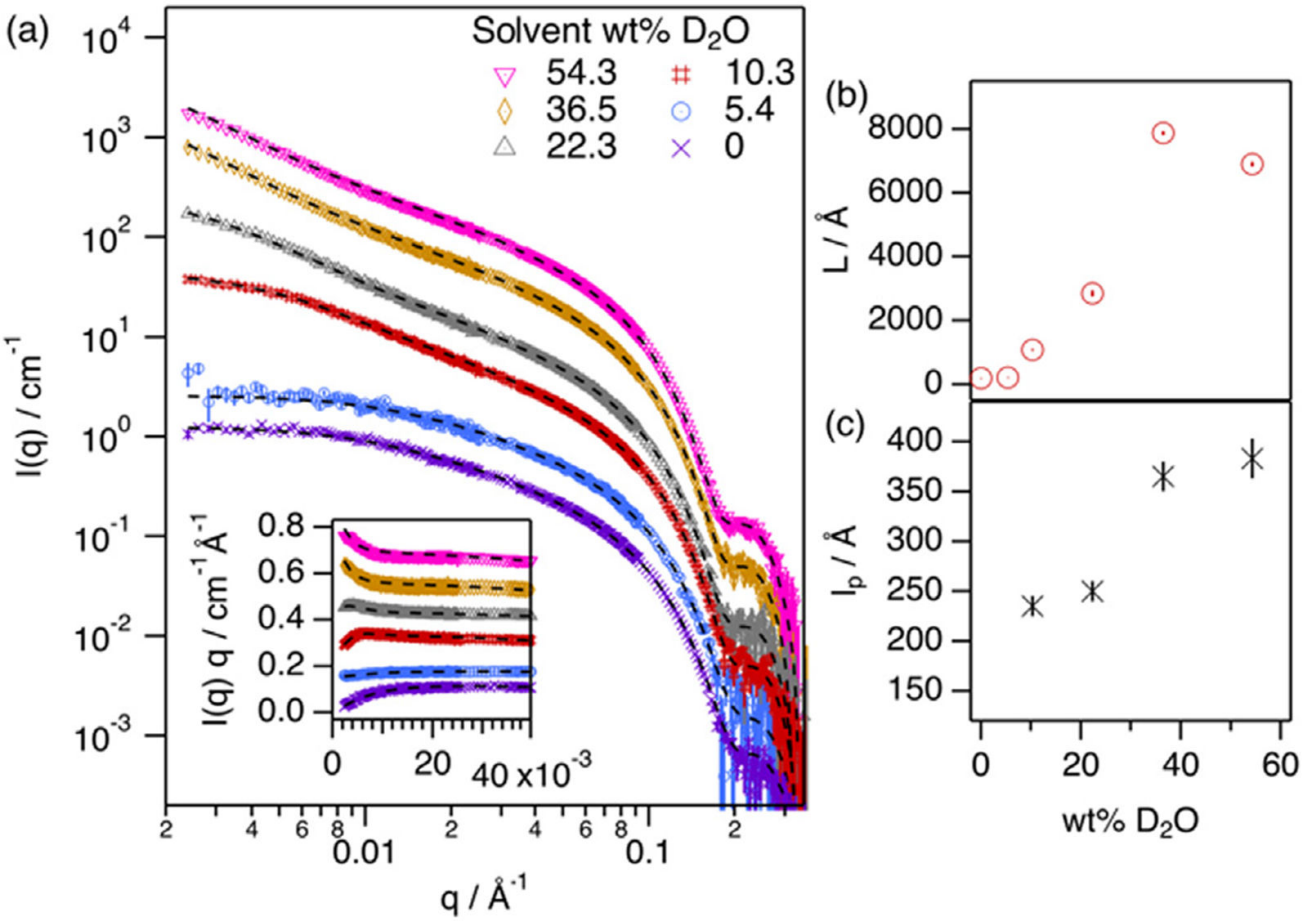
(a) SANS results and best fits of 40 mM h-C_16_TAC at h-ChSal/h-C_16_TAC = 1 in 1:2:n d-ChCl:d-Glyc:D_2_O at different water contents (as shown in the plot legend), where h and d stand for protiated and deuterated compounds. Fits are presented as black dashed lines. Measurements were performed at 50 °C on the vSANS instrument at NCNR (US). Fits are presented as black dashed lines. The inset presents the Holtzer plots (I(q) q vs q) of the SANS data presented in (a). Data and fits have been offset for clarity. Structural parameters derived from the model–based analysis using uniform bodies are presented at different h-ChSal/h-C_16_TAC: (b) contour length (L) and (c) persistence length (l_p_) [32]. Due to the limited q-range of the SANS measurements, the largest dimension which could be probed was ≈3000 Å Thus, values larger than that are an estimation of the elongation that resulted from the fitting approach. All error bars represent one standard deviation and in some cases are smaller than the symbols.

Also, the effect of solvent hydration on the micellization of this surfactant-hydrotrope system was investigated, as water further provides a possibility to tune the physicochemical characteristics of the solvent. Fig. 4 shows these SANS data, best fits (modelled as uniform scatterers) and structural parameters obtained from those fits.

At a constant surfactant-to-hydrotrope ratio of 1 (40 mM C_16_TAC), the addition of water is seen to have a strong effect on micelle morphology (See Fig. 4b). Whilst small variations are observed in the size of the cross-section of the micelle, the contour length of the micelle increases with the addition of water, except for 54.3% by weight (wt%) D_2_O that shows a 15% decrease in L compared to that for 36.5 wt% D_2_O. At low water content (up to 5.4 wt% D_2_O, as measured here) micelles behave as relatively short, stiff cylinders. The addition of more water results in significant growth of the micelles. This phenomenon, intrinsically associated with the imbibition of more hydrotropes into the micelle, can be explained again through the solubility of hydrotropes in the solvent. As the concentration of water increases, the solubility of hydrotropes (relatively hydrophobic) decreases in a more polar environment. This results in a more significant partitioning of the salt towards the hydrophobic domains of the system (micelle core) and, thus, the observed micellar growth. A reversion in this trend is seen at high water content, 54.3 wt% D_2_O, as the micelle length slightly decreases. Interestingly, this same trend is observed for the system in the absence of salt. This could mean that the reversion in the growth is driven by changes in surfactant-solvent interactions (See Fig. S14 and Table S7), which in turn may relate to transitions in solvent structure upon water addition [17].

As with the contour length, the presence of water in the system induces changes in micellar flexibility. When micelles are sufficiently long in the hydrated DES, they become semiflexible aggregates with persistence lengths that increase with water content, as observed in the Holtzer plot (See Fig. 4 inset). The l_p_ values obtained for micelles in DES:water systems increase with water content and, at the highest water content (54.3 wt% D_2_O), these are similar to those observed in aqueous solutions of ionic surfactants [39], and higher than those for WLM in pure DES (See Fig. 2). This effect could be explained by the electrostatic contribution to the micelle flexibility. As water content increases the permittivity of the solvent, electrostatic repulsion becomes more prominent at higher water contents and, as such, the electrostatic contribution to micelle rigidity increases [37,40].

### 3.2. Discussion

The self-assembly of surfactants in DES has previously been studied for several surfactants in (mainly) choline-based DES, showing that a variety of structures are observed [18]. The self-assembly of alkyltrimethylammonium bromide in 1:2 choline chloride:glycerol resulted in the formation of globular micelles, with certain similarities to those in water [20]. When the same surfactants are dissolved in a carboxylic acid-based DES, i.e. 1:1 choline chloride:malonic acid, the resulting structures are significantly more elongated than in the case of the glycerol-based DES [26]. This demonstrated that the micellization of surfactants in DES can be tuned through changes in the solvent properties. As such, the negative charge spread of 1:1 choline chloride:malonic acid, possibly due to the deprotonation of the carboxylic moieties, resulted in a reduction of the charge density at the micelle interface and the subsequent micelle growth [26]. Although elongated micelles were reported then, the maximum length of these was *ca.* 600 Å, and no evidence of the formation of WLM in DES has been previously reported, despite the theoretical high ionic strength of the solvent.

As such, it was hypothesized that stronger surfactant-salt interactions were required to promote the formation of WLM in DES. A recent investigation showed that the presence of hydrotropes promoted the formation of WLM in organic polar solvents (i.e. glycerol, ethylene glycol, and formamide) [41]. The co-assembly of salt and surfactant was attributed to the relatively strong cohesive forces within these solvents, where the salt binds to the micelle and modifies the packing parameter of the monomer in a similar mechanism to that observed in aqueous solution [2]. The cohesive forces within the solvent are also expected to drive the surfactant-hydrotrope co-assembly in DES, as the glycerol-based DES shows similar behavior to neat glycerol [42].

Our contrast variation SANS experiments indeed confirmed that the binding of the hydrotrope to the micelle induces micelle growth in neat and hydrated DES. Interestingly, sodium salicylate was found to be insoluble in DES and, thus, it was necessary to replace the sodium cation by choline to increase the solubility of the hydrotrope [30]. The addition of this salt to the micellar solution resulted in a significant increase in micelle elongation, which became more pronounced at salt-to-surfactant rations above 1 where the formation of WLM was observed. Additionally, micellar growth is also observed at higher hydration levels of the DES, suggesting that the solubility of the salt is decreased in the DES-water systems and the hydrotrope more strongly bound to the micelles in the presence of water. Such a micellar growth is not observed in the absence of salt. Furthermore, the flexibility of the WLM is altered in DES, where neat DES shows a significantly lower persistence length than that observed in aqueous solutions [33]. The addition of water increases micelle stiffness as the persistence length increases in hydrated DES compared to neat DES. These observations were attributed to the charge density of the solvent, which provided sufficient screening to reduce the electrostatic contribution to the intramicellar interaction and, therefore, to the micelle rigidity [37].

## 4. Conclusions

In summary, the interaction between a choline-based hydrotrope, choline salicylate, with a cationic surfactant on micelle morphology has been investigated. In contrast to previous investigations where surfactants were shown to self-assemble into globular or moderately elongated micelles [20,26], it is here shown that flexible worm-lilke micelles are formed in a deep eutectic solvent. Contrast variation small-angle neutron scattering showed an increased elongation of the micelles with the addition of more salt, particularly at salt-to-surfactant ratios above 2. The change in elongation was attributed to the electrostatic screening between the salt and the surfactant headgroup, and the imbibition of the solvophobic domain of the hydrotrope into the micelle core. This mechanism shows certain similarities to those observed in aqueous solutions and organic polar solvents [7,41]. The formation of hybrid deep eutectic solvent:water systems also affects micellization, where at a constant salt-to-surfactant ratio the micelles grow in length and become stiffer. These findings show for the first time the co-assembly of surfactants and hydrotropes in deep eutectic solvents, resulting in the formation of worm-like micelles and opening up new possibilities in the application of these neoteric solvents in formulation technologies, and as rheological modifiers and responsive materials.

## Supplementary Material

Supp1

## References

[1] Ben-Shaul A, May S, Molecular Packing in Cylindrical Micelles, in: Zana R, Kaler EW (Eds.), Giant Micelles, Taylor and Francis, New York, 2007, pp. 41–79.

[2] Dreiss CCA, Wormlike micelles: where do we stand? Recent developments, linear rheology and scattering techniques, Soft Matter. 3 (8) (2007) 956–970. 32900044 10.1039/b705775j

[3] Larsson J, Sanchez-Fernandez A, Mahmoudi N, Barnsley LC, Wahlgren M, Nylander T, Ulvenlund S, Effect of the anomeric configuration on the micellization of hexadecylmaltoside surfactants, Langmuir 35 (43) (2019) 13904–13914. 31566987 10.1021/acs.langmuir.9b01960

[4] Lin Y, Han X, Huang J, Fu H, Yu C, A facile route to design pH-responsive viscoelastic wormlike micelles: Smart use of hydrotropes, J. Colloid Interface Sci 330 (2) (2009) 449–455. 19059596 10.1016/j.jcis.2008.10.071

[5] Li Q, Wang X, Yue X, Chen X, Wormlike micelles formed using Gemini surfactants with quaternary hydroxyethyl methylammonium headgroups, Soft Matter 9 (40) (2013) 9667–9674. 26029776 10.1039/c3sm51722e

[6] Kelly EA, Houston JE, Evans RC, Probing the dynamic self-assembly behaviour of photoswitchable wormlike micelles in real-time, Soft Matter 15 (6) (2019) 1253–1259. 30418450 10.1039/c8sm01948g

[7] Hassan PA, Fritz G, Kaler EW, Small angle neutron scattering study of sodium dodecyl sulfate micellar growth driven by addition of a hydrotropic salt, J. Colloid Interface Sci 257 (1) (2003) 154–162. 16256467 10.1016/s0021-9797(02)00020-6

[8] Magid LJ, Li Z, Butler PD, Flexibility of elongated sodium dodecyl sulfate micelles in aqueous sodium chloride: a small-angle neutron scattering study, Langmuir 16 (26) (2000) 10028–10036.

[9] Kuperkar K, Abezgauz L, Danino D, Verma G, Hassan PA, Aswal VK, Varade D, Bahadur P, Viscoelastic micellar water/CTAB/NaNO(3) solutions: rheology, SANS and cryo-TEM analysis, J. Colloid Interface Sci 323 (2) (2008) 403–409. 18486141 10.1016/j.jcis.2008.04.040

[10] Das N, Cao H, Kaiser H, Warren GT, Gladden JR, Sokol PE, Shape and size of highly concentrated micelles in CTAB/NaSal solutions by Small Angle Neutron Scattering (SANS), Langmuir 28 (33) (2012) 11962–11968. 22827161 10.1021/la2022598

[11] Berret JF, Appell J, Porte G, Linear rheology of entangled wormlike micelles, Langmuir 9 (11) (1993) 2851–2854.

[12] Jerke G, Pedersen JS, Egelhaaf SU, Schurtenberger P, Flexibility of Charged and Uncharged Polymer-like Micelles, Langmuir 14 (21) (1998) 6013–6024.

[13] Smith EL, Abbott AP, Ryder KS, Deep eutectic solvents (DESs) and their applications, Chem. Rev 114 (21) (2014) 11060–11082. 25300631 10.1021/cr300162p

[14] Hammond OS, Bowron DT, Edler KJ, Liquid structure of the choline chloride-urea deep eutectic solvent (reline) from neutron diffraction and atomistic modelling, Green Chem. 18 (9) (2016) 2736–2744.

[15] El Achkar T, Fourmentin S, Greige-Gerges H, Deep eutectic solvents: An overview on their interactions with water and biochemical compounds, J. Mol. Liq 288 (2019) 111028.

[16] Dai Y, Witkamp GJ, Verpoorte R, Choi YH, Tailoring properties of natural deep eutectic solvents with water to facilitate their applications, Food Chem. 187 (2015) 14–19. 25976992 10.1016/j.foodchem.2015.03.123

[17] Hammond OS, Bowron DT, Edler KJ, The Effect of Water upon Deep Eutectic Solvent Nanostructure: An Unusual Transition from Ionic Mixture to Aqueous Solution, Angew. Chem. Int. Ed. Engl 56 (33) (2017) 9782–9785. 28480595 10.1002/anie.201702486 PMC5596335

[18] Warr GG, Atkin R, Solvophobicity and amphiphilic self-assembly in neoteric and nanostructured solvents, Curr. Opin. Colloid Interface Sci 45 (2020) 83–96.

[19] Arnold T, Jackson AJ, Sanchez-Fernandez A, Magnone D, Terry AE, Edler KJ, Surfactant behavior of sodium dodecylsulfate in deep eutectic solvent choline chloride/urea, Langmuir 31 (47) (2015) 12894–12902. 26540438 10.1021/acs.langmuir.5b02596

[20] Sanchez-Fernandez A, Arnold T, Jackson AJ, Fussell SL, Heenan RK, Campbell RA, Edler KJ, Micellization of alkyltrimethylammonium bromide surfactants in choline chloride:glycerol deep eutectic solvent, PCCP 18 (48) (2016) 33240–33249. 27896342 10.1039/c6cp06053f

[21] Sanchez-Fernandez A, Moody GL, Murfin LC, Arnold T, Jackson AJ, King SM, Lewis SE, Edler KJ, Self-assembly and surface behaviour of pure and mixed zwitterionic amphiphiles in a deep eutectic solvent, Soft Matter. 14 (26) (2018) 5525–5536. 29926037 10.1039/c8sm00755a

[22] Li Q, Tong K, Qiu J, Yan M, Tian Q, Chen X, Yue X, Molecular packing of surface active ionic liquids in a deep eutectic solvent: a small angle X-ray scattering (SAXS) study, Soft Matter 15 (25) (2019) 5060–5066. 31180406 10.1039/c9sm00760a

[23] Mukerjee P, The nature of the association equilibria and hydrophobic bonding in aqueous solutions of association colloids, Adv. Colloid Interface Sci 1 (3) (1967) 242–275.

[24] Perche T, Auvray X, Petipas C, Anthore R, Rico-Lattes I, Lattes A, Small Angle Neutron Scattering Study of the Micellization of Sodium Dodecyl Sulfate in Formamide, Langmuir 13 (6) (1997) 1475–1480.

[25] Sanchez-Fernandez A, Edler KJ, Arnold T, Heenan RK, Porcar L, Terrill NJ, Terry AE, Jackson AJ, Micelle structure in a deep eutectic solvent: a small-angle scattering study, PCCP 18 (20) (2016) 14063–14073. 27157993 10.1039/c6cp01757f

[26] Sanchez-Fernandez A, Hammond OS, Jackson AJ, Arnold T, Doutch J, Edler KJ, Surfactant-solvent interaction effects on the micellization of cationic surfactants in a carboxylic acid-based deep eutectic solvent, Langmuir 33 (50) (2017)14304–14314. 29182879 10.1021/acs.langmuir.7b03254

[27] Hayter JB, Penfold J, Determination of micelle structure and charge by neutron small-angle scattering, Colloid Polym. Sci 261 (12) (1983) 1022–1030.

[28] Matthews L, Przybylowicz Z, Rogers SE, Bartlett P, Johnson AJ, Sochon R, Briscoe WH, The curious case of SDS self-assembly in glycerol: Formation of a lamellar gel, J. Colloid Interface Sci 572 (2020) 384–395. 32272313 10.1016/j.jcis.2020.03.102

[29] Sawama Y, Nakano A, Matsuda T, Kawajiri T, Yamada T, Sajiki H, H-D exchange deuteration of arenes at room temperature, Org. Process Res. Dev 23 (4) (2019) 648–653.

[30] Kukawka R, Czerwoniec P, Lewandowski P, Pospieszny H, Smiglak M, New ionic liquids based on systemic acquired resistance inducers combined with the phytotoxicity reducing cholinium cation, New J. Chem 42 (14) (2018) 11984–11990.

[31] Jae Hie M.C. Doucet; Gervaise Alina; Jurrian Bakker; Wim Bouwman; Paul Butler; Kieran Campbell; Miguel Gonzales; Richard Heenan; Andrew Jackson; Pavol Juhas; Stephen King; Paul Kienzle; Jeff Krzywon; Anders Markvardsen; Torben Nielsen; Lewis O’Driscoll; Wojciech Potrzebowski; Ricardo Ferraz Leal; Tobias Richter; Piotr Rozycko; Tim Snow; Adam Washington, SasView version 4.2.2, 2019. 10.5281/zenodo.2652478.

[32] Pedersen JS, Analysis of small-angle scattering data from colloids and polymer solutions: modeling and least-squares fitting, Adv. Colloid Interface Sci 70 (1997) 171–210.

[33] Magid LJ, Han Z, Li Z, Butler PD, Tuning Microstructure of Cationic Micelles on Multiple Length Scales: The Role of Electrostatics and Specific Ion Binding†, Langmuir 16 (1) (2000) 149–156.

[34] Pedersen JS, Schurtenberger P, Scattering functions of semiflexible polymers with and without excluded volume effects, Macromolecules 29 (23) (1996) 7602–7612.

[35] Mukerjee P, Size distribution of small and large micelles. Multiple equilibrium analysis, J. Phys. Chem 76 (4) (1972) 565–570.

[36] Kotlarchyk M, Chen SH, Analysis of small angle neutron scattering spectra from polydisperse interacting colloids, J. Chem. Phys 79 (5) (1983) 2461–2469.

[37] Appell J, Porte G, Poggi Y, Quantitative estimate of the orientational persistence length of flexible elongated micelles of cetylpyridinium bromide, J. Colloid Interface Sci 87 (2) (1982) 492–499.

[38] Hammons JA, Zhang F, Ilavsky J, Extended hierarchical solvent perturbations from curved surfaces of mesoporous silica particles in a deep eutectic solvent, J. Colloid Interface Sci 520 (2018) 81–90. 29529464 10.1016/j.jcis.2018.02.078 PMC5991083

[39] Chen WR, Butler PD, Magid LJ, Incorporating intermicellar interactions in the fitting of SANS data from cationic wormlike micelles, Langmuir 22 (15) (2006) 6539–6548. 16830995 10.1021/la0530440

[40] Lauw Y, Leermakers FA, Stuart MA, Persistence length of wormlike micelles composed of ionic surfactants: self-consistent-field predictions, J. Phys. Chem. B 111 (28)(2007) 8158–8168. 17580859 10.1021/jp071756o

[41] Agrawal NR, Yue X, Feng Y, Raghavan SR, Wormlike micelles of a cationic surfactant in polar organic solvents: extending surfactant self-assembly to new systems and subzero temperatures, Langmuir 35 (39) (2019) 12782–12791. 31525901 10.1021/acs.langmuir.9b02125

[42] Faraone A, Wagle DV, Baker GA, Novak EC, Ohl M, Reuter D, Lunkenheimer P, Loidl A, Mamontov E, Glycerol hydrogen-bonding network dominates structure and collective dynamics in a deep eutectic solvent, J. Phys. Chem. B 122 (3) (2018) 1261–1267. 29336157 10.1021/acs.jpcb.7b11224

