# Supplementary material for: Complex by design: Hydrotrope-induced micellar growth in deep eutectic solvents
Source: J Colloid Interface Sci. Author manuscript; Available in PMC 2023 Aug 30. (PMC10466478; doi:10.1016/j.jcis.2020.07.077)
Supplement: Supp1 [file NIHMS1918305-supplement-Supp1.pdf]

Supplementary Data for:

Complex by design: hydrotrope-induced micellar growth in deep eutectic solvents

Adrian Sanchez-Fernandez,[a]* Anna E. Leung,[b] Elizabeth G. Kelley,[c] Andrew J. Jackson[b,d]

[a]*Food Technology, Engineering and Nutrition, Lund University, Box 124, 221 00 Lund, Sweden. *E-mail: adrian.sanchez-fernandez@food.lth.se*
[b]*European Spallation Source, Box 176, 221 00 Lund, Sweden.*
[c]*The NIST Center for Neutron Research, National Institute of Standards and Technology, Gaithersburg, Maryland 20899-8562, USA.*
[d]*Division of Physical Chemistry, Lund University, Box 124, 221 00 Lund, Sweden*

Materials and methods

Note: The presented uncertainties throughout the Supplementary Data are equal to one standard deviation.

Materials

Table S1 presents the mole ratios prepared and the equivalent wt% of water for each hydrated solvent.

Table S1 Series of sample compositions that were used in the experiments containing hydrated DES.

Water mole eq. n	Water / mol%	Water / wt%
1	25.0	5.4
2	40.0	10.3
5	62.5	22.3
10	76.9	36.5
20	87.0	54.3

Synthesis and characterisation of choline salicylate

Choline chloride-d$_9$ and sodium salicylate were used as starting reactants. D_2O (99.8% isotopic purity), Pt/C (5% wt.), choline chloride and NaOH were purchased from Sigma-Aldrich.

NMR spectra were recorded on a Varian Unity INOVA NMR spectrometer with a 5 mm $^{1}H/^{13}C$ auto-switchable gradient-probe, operating at 400 MHz for ^{1}H and 100 MHz for ^{13}C nuclei. ^{13}C NMR spectra were ^{1}H-decoupled but not ^{2}H-decoupled. Spectra were recorded at 298 K. Chemical shifts, expressed in parts per million (ppm), were referenced to residual solvent signals. Quantitative ^{1}H NMR spectra used a relaxation delay of 57 seconds. Mass spectra were recorded on an Agilent 1260 Infinity II system coupled with an Agilent InfinityLab LC/MSD XT. The column was by-passed and the sample was pumped directly into the mass spectrometer. The overall deuteration incorporation across the molecule was calculated by mass spectrometry using the relative distribution of the different isotopologues. This calculation accounted for the ^{13}C natural abundance by subtracting this contribution from the peak area of each M+1 isotopologue.

Choline salicylate

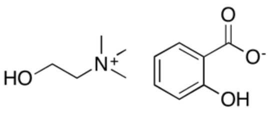

Using a modification of a literature procedure,[1] a solution of sodium salicylate (7.01 g, 43.8 mmol) in anhydrous methanol (85 mL) was added to a solution of choline chloride (6.10 g, 43.7 mmol) in anhydrous methanol (85 mL) and the mixture was stirred for one hour at room temperature. The solvent was removed under reduced pressure and the resulting residue was dried at 40 °C under vacuum overnight. Acetone (2 mL) was added to effect precipitation of the sodium chloride by-product and the suspension was filtered through a syringe filter (PTFE with a 0.2 µm membrane). The acetone was removed from the solution under reduced pressure and the resulting viscous oil was freeze-dried to afford a clear product (9.16 g, 87% yield). ^{1}H NMR (DMSO-d_6, 400 MHz, 57 s relaxation delay) δ 3.11 (s, 9H), 3.34 (br s, 1H, •0.5H_2O), 3.41 (m, 2H), 3.85 (m, 2H), 5.54 (m, 1H), 6.55-6.60 (complex, 2H), 7.11 (m, 1H), 7.64 (dd, J = 7.6, 1.7 Hz, 1H). ^{13}C NMR (DMSO-d_6, 100 MHz) δ 53.14, 53.18, 53.22, 55.2, 67.1 (t, J = 2.7 Hz), 115.9, 116.3, 120.4, 130.1, 131.6, 162.6, 171.9. NMR spectra are presented in Figures S1 and S2.

Note: Yu and co-workers describe this as a partial hydrate based on elemental analysis: $C_{12}H_{19}NO_4$•0.85 H_2O;[2] our ^{1}H NMR spectroscopic data is in agreement with this: $C_{12}H_{19}NO_4$•0.50 H_2O.

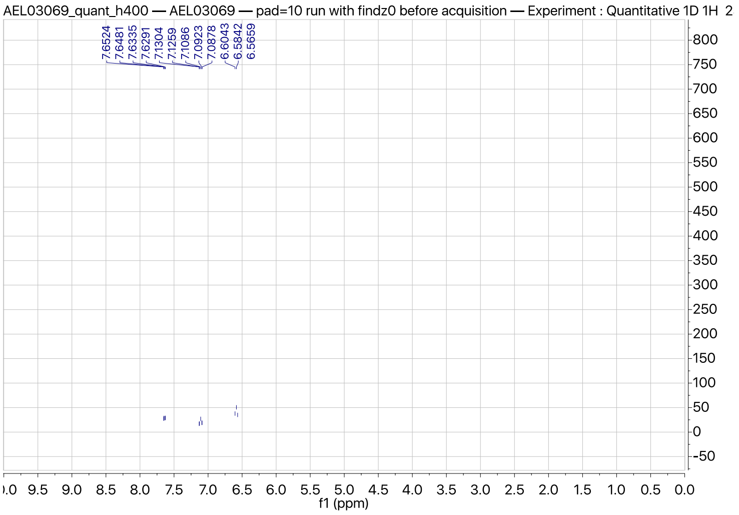

Figure S1 ^{1}H NMR spectrum of choline salicylate (DMSO-d_6, 400 MHz, 57 second relaxation delay).

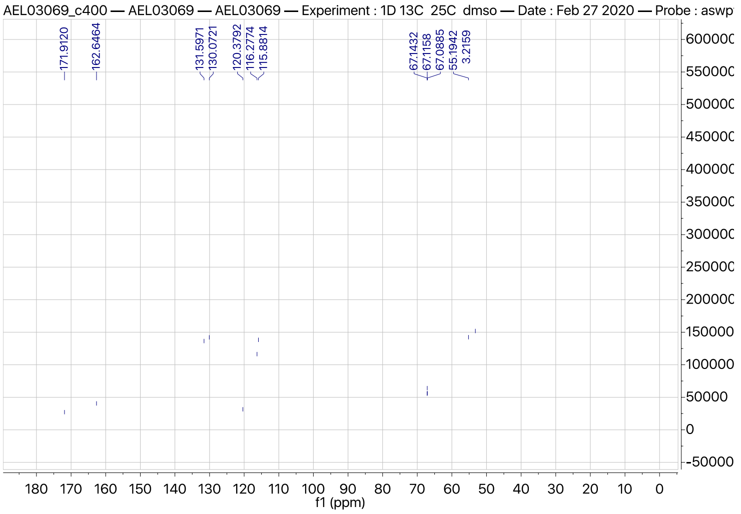

Figure S2 ^{13}C NMR spectrum of choline salicylate (DMSO-d_6, 100 MHz).

Salicylic acid-d_4

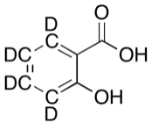

Using a modification of a literature procedure,[3] salicylic acid (4.26 g, 30.8 mmol), Pt/C (5% wt., 3.60 g, 923 µmol), isopropanol (61.5 mL) and D_2O (123 mL) were added to a 500 mL round-bottom flask. The flask was evacuated and then filled with argon ten times and then sealed with a suba seal and stirred at room temperature for four days. The mixture was filtered to remove the catalyst and the catalyst was washed with ethyl acetate (50 mL) and H_2O (7.5 mL). The filtrate was extracted with ethyl acetate (3 x 30 mL) and the combined organic fractions were dried ($MgSO_4$), filtered and concentrated to afford deuterium-labelled salicylic acid-d_4 (3.64 g, 83% yield, 90% of non-exchangeable hydrogen present as deuterium by mass spectrometric analysis). ^{1}H NMR (DMSO-d_6, 400 MHz) δ 6.91-6.96 (complex, residual), 7.51 (br s, residual), 7.79 (m, residual). ^{13}C NMR (DMSO-

d_6, 100 MHz) δ 112.9 (m), 117.0 (m), 118.6 (m), 119.1 (m), 130.3 (m), 135.5 (m), 161.1 (s), 171.9 (s). MS (ESI–) *m/z* calculated for $C_7H_2D_4O_3$ $[M–H]^–$ as 141.1; found: 141.1 (most abundant isotopologue). Isotope distribution: d_1 0.4%; d_2 4.8%; d_3 28.6%; d_4 66.2%. NMR spectra are presented in Figures S3, S4 and S5, and ESI-MS results are included in Figure S6.

Note: ^{13}C NMR data is complicated by the coupling of ^{13}C-^{2}H; spectrum is included below (Figures S4 and S5).

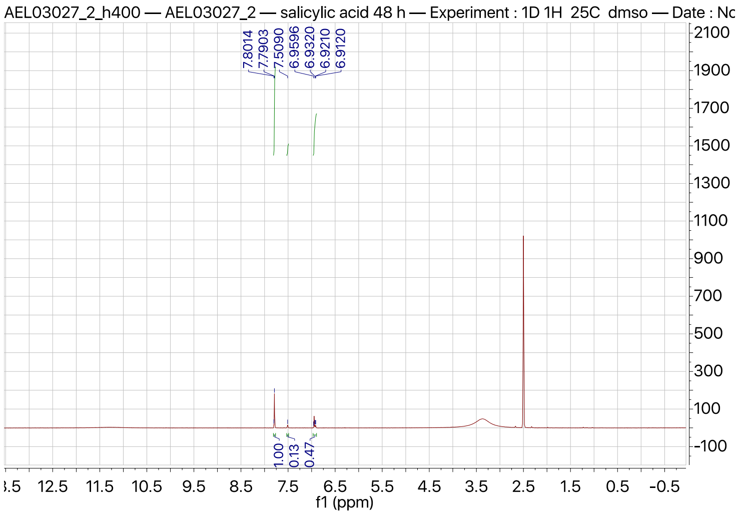

Figure S3 ^{1}H NMR spectrum of salicylic acid-$d_{4(90\%)}$ (DMSO-d_6, 400 MHz).

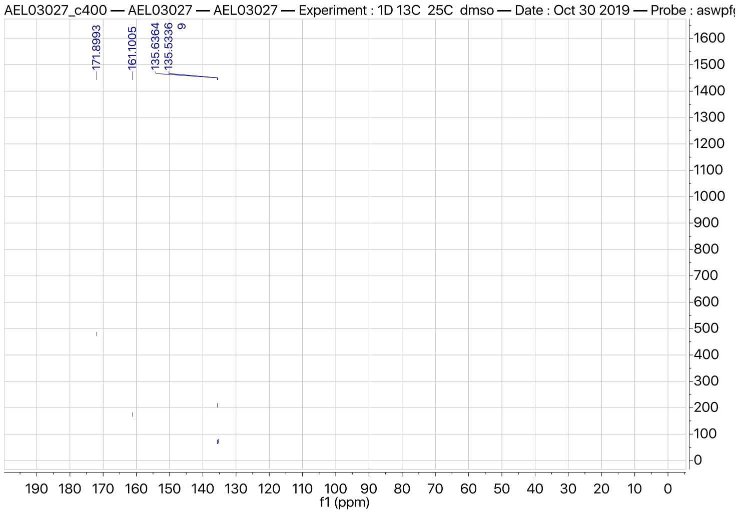

Figure S4 ^{13}C NMR spectrum of salicylic acid-$d_{4(90\%)}$ (DMSO-d_6, 100 MHz).

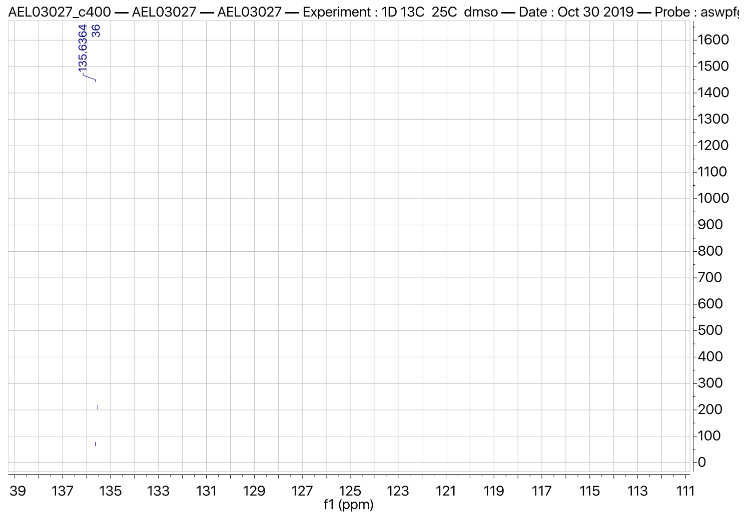

Figure S5 Expansion of ^{13}C NMR spectrum of salicylic acid-$d_{4(90\%)}$ (DMSO-d_6, 100 MHz).

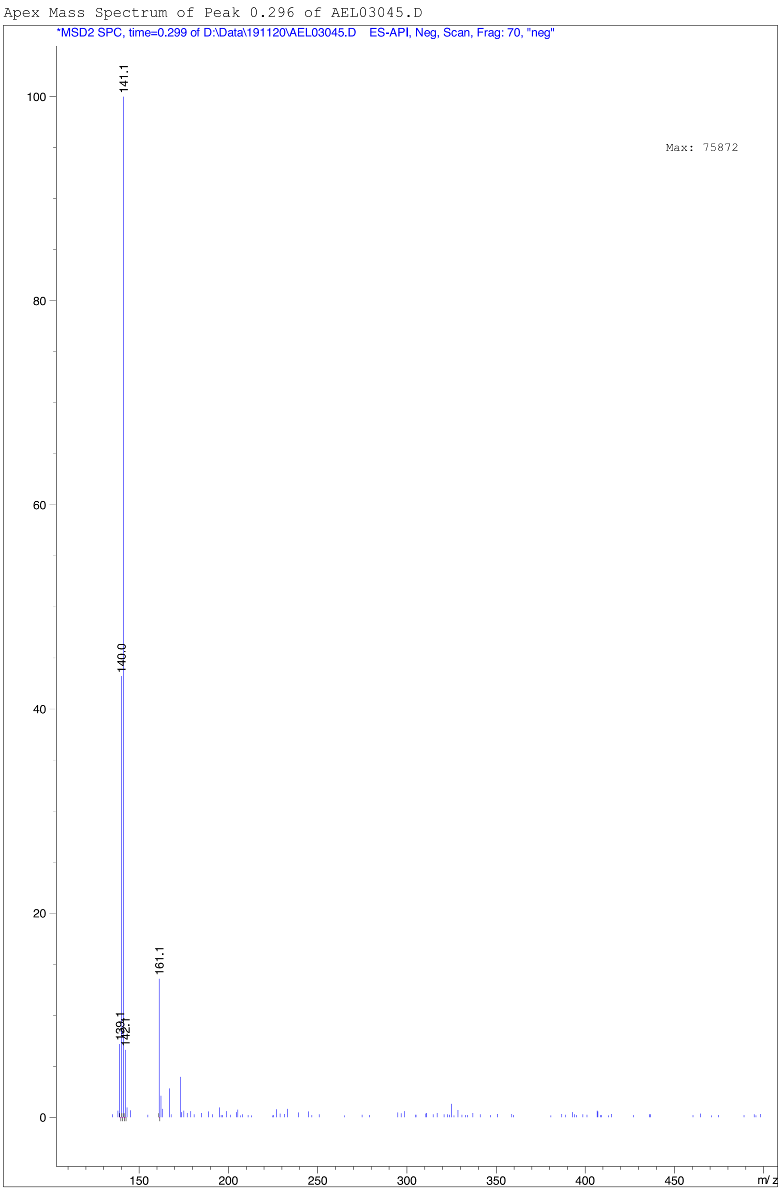

Figure S6 Mass spectrum (ESI–) of salicylic acid-d_4: m/z calculated for $C_7H_2D_4O_3$ $[M–H]^–$ as 141.1; found: 141.1 (most abundant isotopologue).

Sodium salicylate-d_4

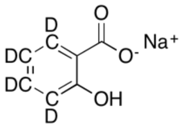

Salicylic acid-d_4 (90% D, 1.64 g, 11.5 mmol), D_2O (6 mL) and NaOD (40% w/w in D_2O, 1.18 g, 11.5 mmol) were heated until the D_2O had evaporated. The resulting solid was washed with diethyl ether (20 mL), then suspended between water (30 mL) and diethyl ether (30 mL) in a separating funnel. The aqueous phase was filtered through a syringe filter (PTFE with a 0.2 µm membrane) and then freeze-dried to afford a white solid (1.78 g, 95% yield). ^1H NMR (CD_3OD, 400 MHz) δ 4.86 (br s, 1H), 6.76 (br s, residual), 6.78 (br s, residual), 7.26 (br s, residual), 7.84 (br s, residual). ^{13}C NMR (DMSO-d_6, 100 MHz) δ 115.8 (m), 116.3 (m), 120.0 (m), 130.2 (m), 131.6 (m), 162.3 (s), 172.7 (s). NMR spectra are presented in Figures S7, S8 and S9.

Note: ^{13}C NMR data is complicated by the coupling of ^{13}C-^2H; spectrum is included below (Figures S8 and S9).

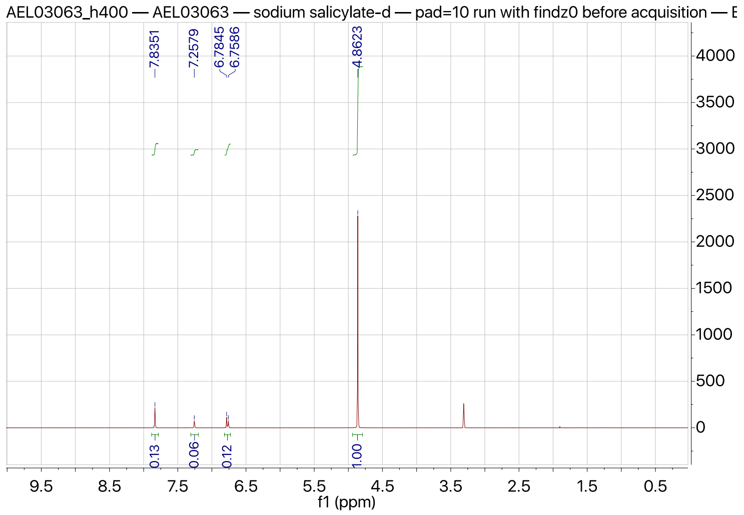

Figure S7. ^1H NMR spectrum of sodium salicylate-$d_{4(90\%)}$ (CD_3OD, 100 MHz).

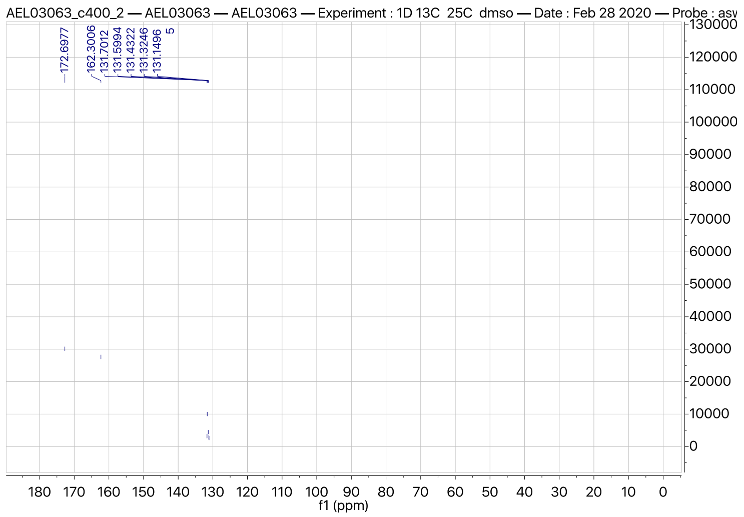

Figure S8. ^{13}C NMR spectrum of sodium salicylate-$d_{4(90\%)}$ (DMSO-d_6, 100 MHz).

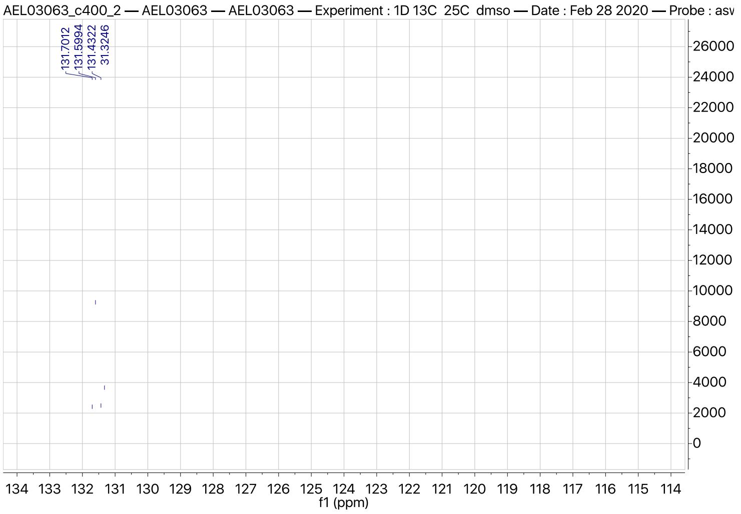

Figure S9. Expansion of ^{13}C NMR spectrum of sodium salicylate-$d_{4(90\%)}$ (DMSO-d_6, 100 MHz).

Choline-d_9 salicylate-d_4

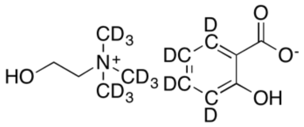

Using a modification of a literature procedure for the unlabelled analogue,[1] a solution of sodium salicylate-d_4 (90% D, 499 mg, 3.04 mmol) in dry methanol (8 mL) was added to a solution of choline chloride-d_9 (451 mg, 3.03 mmol) in dry methanol (8 mL) and the mixture was stirred at room temperature for one hour. The solvent was removed under reduced pressure and the residue was further dried at 40 °C under vacuum overnight. Acetone (2 mL) was added to effect precipitation of the sodium chloride by-product and the suspension was filtered through a syringe filter (PTFE with a 0.2 µm membrane). The acetone was removed from the solution under reduced pressure and the resulting viscous oil was freeze-dried to afford a viscous oil (417 mg, 54% yield). ^{1}H NMR (DMSO-d_6, 400 MHz) δ 3.34 (s, 2 H, •H_2O), 3.39 (m, 2 H), 3.84 (m, 2H), 5.50 (br s, 1 H), 6.56 (br s, residual), 6.59 (br s, residual), 7.10 (br s, residual), 7.64 (br s, residual). ^{13}C NMR (DMSO-d_6, 100 MHz) δ 52.1 (m), 55.1 (s), 66.7 (t), 115.66 (m), 115.74 (m), 120.4 (s), 129.8 (m), 131.2 (m), 162.8 (s), 171.6 (s). NMR spectra are presented in Figures S10, S11 and S12.

Note: Yu and co-workers describe the unlabelled analogue as a partial hydrate based on elemental analysis: $C_{12}H_{19}NO_4$•0.85 H_2O; our ^{1}H NMR spectroscopic data suggests our sample is a monohydrate: $C_{12}H_6D_{13}NO_4$•H_2O.[2]

^{13}C NMR data is complicated by the isotopic labelling and coupling of ^{13}C-^{2}H; spectrum is included below (Figures S11 and S12).

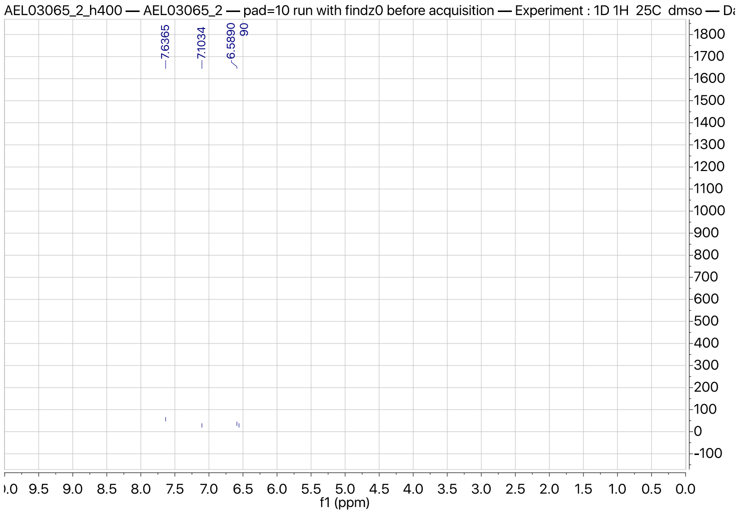

Figure S10. ^{1}H NMR spectrum of choline-$d_{9(98\%)}$ salicylate-$d_{4(90\%)}$ (DMSO-d_6, 400 MHz).

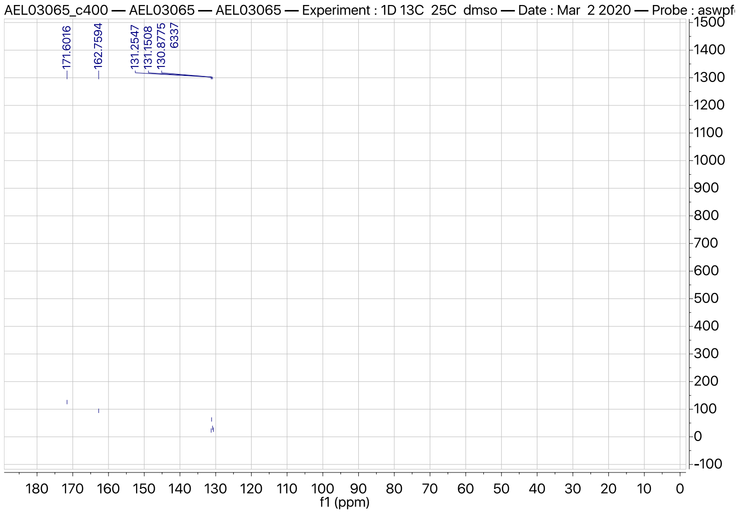

Figure S11. ^{13}C NMR spectrum of choline-$d_{9(98\%)}$ salicylate-$d_{4(90\%)}$ (DMSO-d_6, 100 MHz).

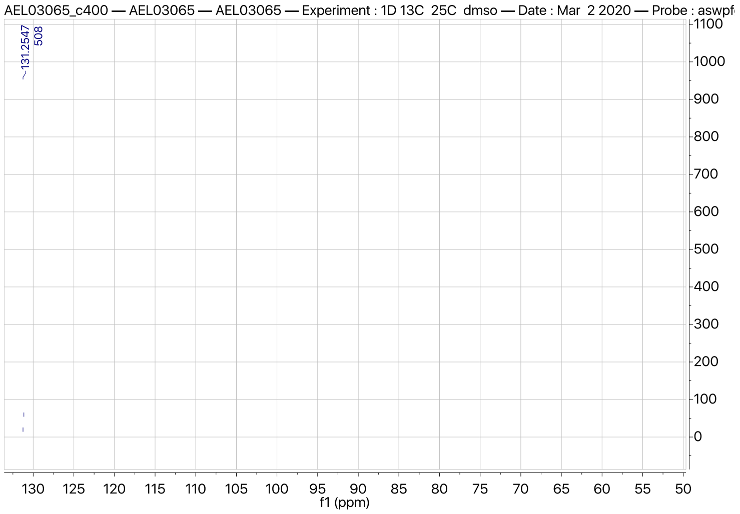

Figure S12. Expansion of ^{13}C NMR spectrum of choline-$d_{9(98\%)}$ salicylate-$d_{4(90\%)}$ (DMSO-d_6, 100 MHz).

Methods

Neutron scattering length densities

The neutron scattering length densities (SLD) of each component of the system was calculated from the neutron scattering length of the unit (b) and the volume it occupies (V_m). These values are presented in Table S2.

Table S2 Scattering lengths, volumes and scattering length densities of each component of the system.

Unit	b^b / fm	V_m / Å^3	SLD / ×10^{-6} Å^{-2}
h-C_{16}	-17.1	458[a]	-0.37
h-ChSal	41.3	350	1.18
d-ChSal	176.7	350	5.04
1:2 d-ChCl:d-Glyc	280.7	435	6.45
1:2:1 d-ChCl:d-Glyc:D_2O	299.9	465	6.45
1:2:2 d-ChCl:d-Glyc:D_2O	319.0	495	6.45
1:2:5 d-ChCl:d-Glyc:D_2O	376.5	585	6.44
1:2:10 d-ChCl:d-Glyc:D_2O	472.2	735	6.43
1:2:20 d-ChCl:d-Glyc:D_2O	663.6	1034	6.42

[a]The volume for surfactant tails were obtained from Tanford equations.[4]
[b]Scattering lengths were calculated as the summation of the neutron scattering lengths of the atoms which form the unit.

Structural parameters of h-C_{16}TAC/h-ChSal micelles in 1:2 d-choline chloride:d-glycerol and 1:2 d-choline chloride:d-glycerol:D_2O

The structural parameters obtained from the SANS data analysis, as presented in the Methods section, for 40 mM h-C_{16}TAC at different h-ChSal concentrations in d-ChCl:d-Glyc, and 40 mM h-C_{16}TAC, 40 mM h-ChSal in 1:2:n d-ChCl:d-Glyc:D_2O at different hydration levels are presented in Table S3 and Table S4, respectively.

Table S3 Structural parameters from the analysis of SANS data presented in Figure 1 of the main text using different mathematical models: ellipsoid (Ell), cylinder (Cyl) or flexible cylinder (FlexCyl).

ChSal/C_{16}TAC	Model	r / Å	L / Å	l_p / Å
0	Ell	21.5±0.4	67.2±0.8	-
0.2	Cyl	19.2±0.3	80.4±0.7	-
0.5	Cyl	19.4±0.2	109±1	-
1	Cyl	19.4±0.3	190±2	-
2	Cyl	20.1±0.2	752±19	-
5	FlexCyl	20.2±0.2	2900±40	166±17
8	FlexCyl	19.7±0.1	8490±330[a]	147±14

Table S4 Structural parameters from the analysis of SANS data presented in Figure 3 of the main text using different mathematical models: ellipsoid (Ell), cylinder (Cyl) or flexible cylinder (FlexCyl).

D_2O wt%	Model	r / Å	L / Å	l_p / Å

0	Cyl	19.4±0.3	190±2	-
5.4	Cyl	19.4±0.2	206±2	-
10.3	FlexCyl	20.5±0.2	1080±20	235±10
22.3	FlexCyl	21.1±0.2	2840±90	250±10
36.5	FlexCyl	21.6±0.2	7870±60[a]	365±15
54.3	FlexCyl	21.9±0.2	6900±70[a]	383±20

[a]Due to the limited q-range of the SANS measurements, the largest dimension which could be probed was ~3000 Å. Thus, this value is an estimation of the elongation that resulted from the fitting approach.

Structural parameters of h-C_{16}TAC/d-ChSal micelles in 1:2 d-choline chloride:d-glycerol

Contrast variation SANS was used to probe the molecular origin of the interaction between the surfactant and the hydrotrope. The structure of the co-assemblies of h-C_{16}TAC and d-ChSal in 1:2 d-ChCl:d-Glyc was determined at the same molar concentrations used for the other contrast (Figure 1 of the main text): (40 mM h-C_{16}TAC, d-ChSal/h-C_{16}TAC=0.2, 0.5, 1, 2, 5 and 8). The SANS data and best fits are presented in Figure 2a, and the structural parameters obtained from those fits are included in Table S5.

Table S5 Structural parameters from the analysis of SANS data presented in Figure 2a using different mathematical models: cylinder (Cyl) or flexible cylinder (FlexCyl).

ChSal/C_{16}TAC	Model	r / Å	L / Å	l_p / Å
1	Cyl	19.2±0.2	184±3	-
2	Cyl	19.3±0.2	642±16	-
5	FlexCyl	19.3±0.2	1080±20	189±8

Effect of choline acetate on the micellization of C_{16}TAC in 1:2 choline chloride:glycerol

In order to study the electrostatic contribution of the hydrotrope-surfactant interaction, the micellar structure of 40 mM h-C_{16}TAC in 1:2 d-ChCl:d-Glyc in the presence of 80 mM h-ChAc was investigated using SANS. Data from the surfactant micelles in the absence of salt and in the presence of ChAc were best fitted using a uniform ellipsoid model. Data from 40 mM h-C_{16}TAC in 1:2 d-ChCl:d-Glyc in the presence of 80 mM h-ChSal, fitted using a cylinder model, is presented for comparison. SANS data and best are presented in Figure 2b. The structural parameters obtained from those fits are presented in Table S6.

Table S6 Structural parameters from the analysis of SANS data of 40 mM h-C_{16}TAC with no salt, 80 mM ChAc and 80 mM ChSal in 1:2 d-ChCl:d-Glyc, as presented in Figure 2b using different mathematical models: ellipsoid (Ell) or cylinder (Cyl).

System	Model	r / Å	L / Å	ϕ / ×10^{-2}
No salt	Ell	21.5±0.4	67.2±0.8	0.27±0.05
80 mM ChAc	Ell	21.7±0.4	68.6±0.8	0.26±0.06
80 mM ChSal	Cyl	20.1±0.2	752±19	0.63±0.03

When comparing the molecular structure of the two salts, it is observed that the anion of ChAc is a methyl carboxylate group and, thus, lacks the phenol group of the hydrotrope, as shown in Figure S13.

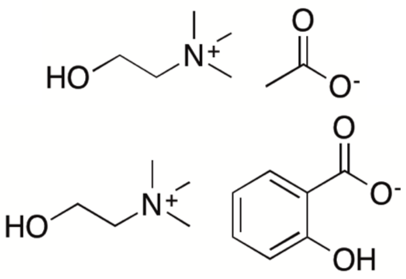

Figure S13 Molecular structure of ChAc (top) and ChSal (bottom).

The results show very similar structural characteristics between the system without salt and with 80 mM ChAc, whilst ChSal promotes micelle growth at the same molar concentration. Furthermore, it is observed that the addition of the hydrotrope increases the volume fraction of micelles (ϕ), a common behaviour observed in the presence of interacting salts which reduce the surfactant CMC and adsorb to the micelle.[5] Therefore, it can be concluded that the interaction of ChAc with the surfactant is negligible, and as such the hydrophobic moiety of the hydrotrope is required to induce the structural transitions presented here.

Micellization of C_{16}TAC in 1:2:n choline chloride:glycerol:water

The behaviour of the surfactant in the absence of salt has been studied to establish a comparison with the micellization in the presence of salt. The micellar structure of 40 mM h-C_{16}TAC in 1:2:n d-ChCl:d-Glyc:D_2O at different water contents was investigated using SANS. Figure S14 presents the SANS data and best fits using a uniform ellipsoid model. The structural parameters obtained from those fits are presented in Table S7.

Additionally, the micellar shape of electrostatically interacting C_{16}TAC micelles in aqueous solution was determined for comparison. These data were fitted using a uniform ellipsoid form factor and a mean spherical approximation (MSA) structure factor, as previously reported.[6]

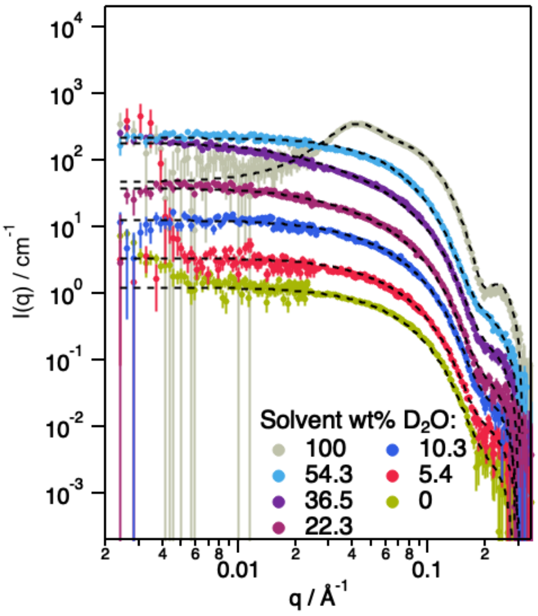

Figure S14 SANS results and best fits of 40 mM h-C_{16}TAC in 1:2:n d-ChCl:d-Glyc:D_2O at different water contents (as shown in the plot legend) where h and d stand for protiated and deuterated compounds. Fits using uniform ellipsoid are presented as black dashed lines.

Table S7 Structural parameters from the analysis of SANS data of 40 mM h-C_{16}TAC in 1:2:n d-ChCl:d-Glyc:D_2O at different water contents, as presented in Figure S14, where h and d stand for protiated and deuterated compounds.

D_2O wt%	Model	r / Å	L / Å	ϕ / ×10^{-2}
0	Ell	21.5±0.4	67.2±0.8	0.27±0.05
5.4	Ell	23.5±0.3	65.6±0.4	0.32±
10.3	Ell	22.9±0.5	72.4±0.3	0.53±
22.3	Ell	22.8±0.3	104±1	0.56±
36.5	Ell	22.0±0.2	156±1	0.96±
54.3	Ell	23.0±0.2	100±1	1.1±2
100	Ell×MSA	21.6±0.3	59.6±0.4	1.1±0.1

These results show that the micellization of C_{16}TAC in ChCl:Glyc is solvent dependant. The micellar cross-section shows small variations and these are within instrument resolution. The elongation of the micelles undergoes a non-monotonic change with increasing water content. As solvent hydration is increased, micelles grow in contour length up to 36.5 wt% D_2O, as seen in Table S7. This transition may be related to transitions within the solvent structure as induced by the presence of water, where the presence of dissociated DES components may interact with surfactant headgroups and decrease the charge density at the headgroup region. Interestingly, this behaviour is the opposite to that observed in interacting self-assembly in DES.[5, 7] Above 36.5 wt% D_2O, micelles become shorter. This may be linked to the opposite effect, as an increase in water content will result in lower salt content and higher solvent polarity. Therefore, the charge density in the headgroup region will increase and thus promote shorter micelles. When DES is removed, micelles are strongly interacting globular assemblies, as previously reported.[6]

Disclaimer

Any mention of commercial products in this manuscript are for information only and does not imply endorsement nor recommendation by NIST.

References

[1] R. Kukawka, P. Czerwoniec, P. Lewandowski, H. Pospieszny, M. Smiglak, New ionic liquids based on systemic acquired resistance inducers combined with the phytotoxicity reducing cholinium cation, New J. Chem. 42(14) (2018) 11984-11990.

[2] Y. Yu, X. Lu, Q. Zhou, K. Dong, H. Yao, S. Zhang, Biodegradable Naphthenic Acid Ionic Liquids: Synthesis, Characterization, and Quantitative Structure–Biodegradation Relationship, Chemistry – A European Journal 14(35) (2008) 11174-11182.

[3] Y. Sawama, A. Nakano, T. Matsuda, T. Kawajiri, T. Yamada, H. Sajiki, H–D Exchange Deuteration of Arenes at Room Temperature, Organic Process Research & Development 23(4) (2019) 648-653.

[4] C. Tanford, Micelle shape and size, J. Phys. Chem. 76(21) (1972) 3020-3024.

[5] A. Sanchez-Fernandez, O.S. Hammond, A.J. Jackson, T. Arnold, J. Doutch, K.J. Edler, Surfactant-Solvent Interaction Effects on the Micellization of Cationic Surfactants in a Carboxylic Acid-Based Deep Eutectic Solvent, Langmuir 33(50) (2017) 14304-14314.

[6] J.B. Hayter, J. Penfold, Determination of micelle structure and charge by neutron small-angle scattering, Colloid & Polymer Science 261(12) (1983) 1022-1030.

[7] A. Sanchez-Fernandez, K.J. Edler, T. Arnold, R.K. Heenan, L. Porcar, N.J. Terrill, A.E. Terry, A.J. Jackson, Micelle structure in a deep eutectic solvent: a small-angle scattering study, Phys. Chem. Chem. Phys. 18(20) (2016) 14063-73.
